# Human Sexuality and Adolescence

**DOI:** 10.3389/fpubh.2013.00041

**Published:** 2013-10-07

**Authors:** Joav Merrick, Ariel Tenenbaum, Hatim A. Omar

**Affiliations:** ^1^National Institute of Child Health and Human Development, Jerusalem, Israel; ^2^Health Services, Division for Intellectual and Developmental Disabilities, Ministry of Social Affairs and Social Services, Jerusalem, Israel; ^3^Division of Pediatrics, Hadassah Hebrew University Medical Center, Jerusalem, Israel; ^4^Division of Adolescent Medicine, Kentucky Children’s Hospital, University of Kentucky, Lexington, KY, USA

**Keywords:** sexual behavior, sexuality, adolescent behavior, adolescence, human sexuality

## Introduction

Human sexuality involve sexual attraction to another person, which for the most part is to the opposite sex (heterosexuality), some to the same sex (homosexuality), or some having both (bisexuality) or not being attracted to anyone in a sexual manner (asexuality).

Human sexuality is determined by many factors, like cultural, political, legal, and philosophical aspects of life, but also morality, ethics, theology, spirituality, and religion. Sexuality is as old as mankind and interest in sexual activity is very much related to the onset of puberty and the period of schooling.

Primary care physicians see children and adolescents with a wide variety of gynecologic and sexual needs, though they often have limited training and limited available educational materials to help them. We therefore published a small handbook on pediatric and adolescent gynecology with information for the primary care clinician (1), but in this book we have gathered papers from around the world in order to discuss issues of sexuality from an international perspective.

Children and adolescents must successfully traverse various stages of pubertal and sexual development in order to become well-adjusted adults with established sexual identity, functioning, and ability. Concerns with breast development and menstrual function are common issues with adolescent females. The clinician may be presented with children who have pediatric gynecologic complaints (i.e., vulvar bleeding, itching, or a mass) or an adolescent who needs contraception or pregnancy counseling.

## Youth Risk Behavior Survey

The United States 2005 Youth Risk Behavior Survey (YRBS) (2) noted that 46.8% of all high school students have had sexual (coital) experience, with a range of 43.0% for Whites, 51.0% for Hispanics, and 67.6% for African-American youth. These millions of sexually active youth produce about 900,000 pregnancies and over six million sexually transmitted diseases (STDs) each year, 1 in 10 of adolescent females becomes pregnant each year, 90% of which were unintended. In adolescents, 14% of the pregnancies end in miscarriages, 35% in abortions, and 51% in live births. Pregnancy rates and STD numbers are also significant around the world, including European countries.

Studies note (3) that youth (age 15–19 years) have the highest rates of chlamydia, gonorrhea cervicitis (endocervicitis), syphilis, and hospitalizations due to pelvic inflammatory disease (PID). Worldwide, those individuals 15–29 years of age have the highest STD risk. Increased risk of youth for STDs is due to high sexual activity rates, multiple sex partners, use of sex and drugs concomitantly, immature cervix (sensitive transition zone cells exposed to the vaginal environment), “adolescence myth” thinking (i.e., no harm will ever come to one, despite high risk behavior), and difficulty in dealing with the medical system for prevention, screening, diagnosis, and treatment. The cost in terms of preventable health care spending is staggering and the complications of STDs are severe, especially for females; these include PID, chronic pelvic pain, ectopic (tubal) pregnancy, and poor pregnancy outcomes, HPV-induced cervical cancer, and others.

In the United States, in 2005 (2), there were 1,225 newly diagnosed cases of HIV/AIDS among adolescents 15–19 years old, and 3,904 among young adults aged 20–24 years (out of 200,000 cases in the United States). There is an estimated one million Americans (4), who are infected with HIV; it is unknown how many are teenagers who will eventually die from HIV in their third or fourth decade of life. Approximately 20% of adults with HIV infection were infected as teenagers and one in four individuals newly diagnosed with HIV infection is under age 22 years. African-Americans are 14% of the American population, yet they bear 28% of the AIDS cases in America. It is estimated that nearly 100,000 women in the United Kingdom develop PID each year. After one episode of STI/STD, 12.8% of young women will suffer from tubal infertility; after three episodes, this figure rises to 75%. However, because most primary care providers do not deal with the complex treatment of AIDS, we will not include a chapter on the subject in this book.

There are approximately three million reported annual cases of abuse in America in those under 18 years of age (5); in 2004 reported cases of maltreatment were subdivided into neglect in 62.4%, physical abuse in 17.5%, sexual abuse in 9.7%, emotional or psychological abuse in 7%, and medical neglect in 2.1% of total cases (1). The epidemiology of rape suggests that, while fewer than one-third of rapes get reported, in three-quarters of cases the perpetrator is known to the woman/victim. The child or adolescent who is subjected to violence and abuse may become an adult with serious sequelae, including diverse mental health disorders. Many cases of physical abuse of children start out as an attempted sexual abuse and the physical injuries happen as the perpetrator is suppressing the victim’s struggle and resistance.

## Conclusion

The shortage of child and adolescent gynecologists in the world has placed considerable strain on primary care clinicians in helping these children and adolescents, who have a wide variety of sexual and gynecologic needs. The inadequate training primary care clinicians often receive in this area is also worsened by the limited number of available books written for them, which was the reason why we did the pediatric and adolescent gynecology book (1).

## References

[1] Omar HA Greydanus HA Tsitsika AK Patel DR Merrick J editors. Pediatric and Adolescent Sexuality and Gynecology. Principles for the Primary Care Clinician. New York: Nova Science (2010).

[2] Eaton DK Kann L Kinchen S Ross J Hawkins J Harris WA Youth risk behavior surveillance—United States, 2005. MMWR Surveill Summ (2006) 55:1–108 16760893

[3] Weinstock H Berman S Cates W Jr Sexually transmitted diseases among American youth: incidence and prevalence estimates, 2000. Perspect Sex Reprod Health (2004) 36:6–10 10.1363/3600604 14982671

[4] Centers for Disease Control and Prevention Monitoring selected national HIV prevention and care objectives by using HIV surveillance data—United States and 6 U.S. dependent areas—2010. HIV Surveillance Supplemental Report (2012); 17(No. 3, part A). Available from: http://www.cdc.gov/hiv/pdf/statistics_2010_HIV_Surveillance_Report_vol_17_no_3.pdf

[5] U.S. Department of Health and Human Services, Administration for Children and Families, Administration on Children, Youth and Families, Children’s Bureau (2012). Child Maltreatment 2011. Available from: http://www.acf.hhs.gov/programs/cb/research-data-technology/statistics-research/child-maltreatment

